# Determinants of Brachial-Ankle Pulse Wave Velocity in Chinese Patients with Rheumatoid Arthritis

**DOI:** 10.1155/2013/342869

**Published:** 2013-07-31

**Authors:** Ping Li, Cheng-xun Han, Cui-li Ma, Jia-long Guo, Bo Liu, Juan Du, Li-qi Bi

**Affiliations:** ^1^Department of Rheumatology and Immunology, China-Japan Union Hospital of Jilin University, Changchun, Jilin 130033, China; ^2^Jilin Province People's Hospital, Changchun, Jilin 130021, China

## Abstract

*Objective*. To investigate the relationship between Brachial-ankle pulse wave velocity (baPWV), and its associated risk factors in Chinese patients with RA. *Methods*. 138 Chinese RA patients and 150 healthy subjects were included. baPWV of all the participants was measured. RA related factors were determined, as well as traditional cardiovascular risk factors. *Results*. baPWV was significant higher in RA group (1705.44 ± 429.20 cm/s) compared to the healthy control group (1386.23 ± 411.09 cm/s) (*P* < 0.001). Compared with low baPWV group, high baPWV group patients were significantly older (*P* = 0.008) and taller (*P* = 0.033). Serum cholesterol (*P* = 0.035), triglycerides (*P* = 0.004), and LDL level (*P* = 0.006) were significantly higher in high baPWV group patients compared with low baPWV group patients. The baPWV of RA patients was positively correlated with age (*r* = 0.439, *P* < 0.001), and serum cholesterol level (*r* = 0.231, *P* = 0.035), serum triglycerides level (*r* = 0.293, *P* < 0.001), serum LDL level (*r* = 0.323, *P* = 0.003). Meanwhile, baPWV negatively correlated with the height of RA patients (*r* = −0.253, *P* = 0.043). Multivariate regression analysis showed that baPWV of RA group was independently associated with age and serum triglycerides level. *Conclusions*. The old age and high level of serum triglycerides may be the major determinants of arterial stiffness in Chinese RA patients.

## 1. Introduction

Rheumatoid arthritis (RA) is a chronic, inflammatory disease of progressive joint destruction. It is associated with increased cardiovascular morbidity and mortality, mostly due to an excess of cardiovascular disease [1–3]. Even after adjustment for traditional cardiovascular risk factors such as hypertension, diabetes mellitus, smoking, and hypercholesterolemia, there is a higher rate of atherosclerosis and cardiovascular events in patients with RA than in healthy subjects. Therefore, it is suggested that additional mechanisms are responsible for the excess cardiovascular risk observed in RA [4, 5]. 

Arterial stiffness has been shown to be an independent predictor of cardiovascular mortality and become a useful index in the prevention and early detection of cardiovascular disease [6, 7]. BaPWV which reflects the stiffness of both central and peripheral muscular arteries has been frequently used as a simple and noninvasive index for assessing arterial stiffness [8–10]. It is the useful tool for identifying a subgroup in the population that is at increased risk for cardiovascular events [11].

The relationship between RA-related factors, as well as traditional cardiovascular risk factors, and the arterial stiffness measured by baPWV has not previously been established in Chinese patients with RA. The aims of this study were to investigate the relationship between arterial stiffness and its associated risk factors in Chinese patients with RA.

## 2. Material and Methods

### 2.1. Patients

A total of 138 RA patients, with 111 of them being women, attending outpatient clinics at China-Japan Union Hospital of Jilin University, were enrolled in this study. All the patients met the American College of Rheumatology 1987 revised criteria for RA [12]. Patients with hypertension, diabetes, heart failure, renal failure (serum creatinine > 1.5 mg/dL), hepatic failure, heart valve disease, and previous myocardial infarction anamnesis and smokers were excluded from the study. 150 age- and sex-matched healthy volunteers were recruited as controls. The individuals of control group had no history of rheumatic disease. The study was approved by the ethics committee of China-Japan Union Hospital and is in accordance with the Helsinki Declaration. 

Information was obtained from all subjects regarding traditional cardiovascular risk factors. Blood pressure (BP) and lipid level were measured. BP was obtained manually in supine position after at least 5 min of rest. The height and weight of the subjects were measured just before examining baPWV. The body mass index (BMI) was expressed as the weight in kilograms divided by squared height in meters (kg/m^2^). Information on current medication use of patients and controls was obtained by questionnaire. The medical records of patients specially focused on disease-modifying antirheumatic drugs (DMARDs), nonsteroidal antiinflammatory drugs (NSAIDs), or corticosteroid usage for 6 months before the study.

### 2.2. Laboratory Analysis

Blood samples were collected after at least 8 hours of fasting. Serum glucose, lipid profile, C-reactive protein (CRP), erythrocyte sedimentation rate (ESR), anticyclic citrullinated peptide (anti-CCP), rheumatoid factor IgM (RF-IgM), RF-IgG, and RF-IgA were checked concurrently in study day. 

### 2.3. Evaluation of RA Disease Activity

Disease activity was evaluated by the Disease Activity Score for 28 joints (DAS28). This utilizes the ESR, visual analogue score (VAS), and the number of tender and swollen joints, from a total of 28 joints assessed as in the following equation [13, 14]:

(1) DAS28=0.56×number  of  tender  joints+0.28×number of  swollen  joints+[0.70×ln⁡(ESR)]+(0.014×VAS).

### 2.4. Measurement of baPWV

After the subjects had rested in a supine position for more than 5 min, the measurement of baPWV was conducted using a waveform analyzer (VP-2000, Colin Co Ltd, Komaki, Japan). Pulse waves were recorded automatically by sensors in the cuffs. The transmission times and distances between the cuffs of arms and legs were recorded, and the baPWV was outputted. The mean of the baPWVs in the left and right sides was used for the analysis. Patients were divided into two subgroups according to mean baPWV value of RA group: those above the mean baPWV value were in the high baPWV group, while those below the mean baPWV value were in the low baPWV group.

### 2.5. Statistical Analysis

The statistical analysis was performed using the SPSS (SPSS ver 17.0 for Window, Chicago, IL, USA) software package. All data were expressed by mean ± SD for normal distributed values and median (interquartile range, IQR) for nonparametric values. Comparisons between the two groups were done by Student's *t*-test or *χ* ^2^ test. Nonparametric data were compared using Mann-Whitney *U* test. Univariate analysis was done to explore relationships between baPWV and other variables by Pearson correlation test for normally distributed data and Spearman Rank correlation test for non-parametric data. Stepwise multiple linear regression analysis was used to assess the independent determinants of increased baPWV. *P* level less than 0.05 was considered statistically significant.

## 3. Results

### 3.1. Clinical Characteristics of Patients and Controls

Characteristics of patients and controls are given in Table 1. Among the traditional cardiovascular risk factors, only triglycerides levels were higher in patients with RA compared with controls (*P* < 0.05). RA patients had higher baPWV than controls (*P* < 0.001). The other factors had no difference between RA patients and controls.

### 3.2. Disease-Related Factors

The mean disease duration was 24 (10.00–102.00) months. 80 (57.97%) patients were RF-IgG positive, 65 (47.10%) were RF-IgA positive, 108 (78.26%) were RF-IgM positive, and 98 (71.01%) were anti-CCP positive. The mean DAS28 was (5.08 ± 1.00). It indicated that disease activity of the RA patients studied was moderate to high. At the time of measurement, 52.90% of patients were treated with NSAIDs and 6.52% with DMARDs. No patient was treated with corticosteroid (Table 2).

### 3.3. Patient Characteristics and Comparisons between Two Subgroups of RA Patients

Table 3 shows comparisons of the demographic and clinical characteristics between two subgroup patients divided according to the mean baPWV value of RA group. Compared with low baPWV group, high baPWV group patients were significantly older (*P* = 0.008) and taller (*P* = 0.033). Serum cholesterol (*P* = 0.035), triglycerides (*P* = 0.004), and LDL level (*P* = 0.006) were significantly higher in high baPWV group patients compared with low baPWV group patients. However, there were no significant differences in RA-related factors between two subgroups (*P* > 0.05).

### 3.4. Correlations between baPWV and Other Variables

Table 4 shows correlations between baPWV and clinical parameter in RA patients. The baPWV of RA patients was positively correlated with age (*r* = 0.439, *P* < 0.001), serum cholesterol level (*r* = 0.231, *P* = 0.035), serum triglycerides level (*r* = 0.293, *P* < 0.001), and serum LDL level (*r* = 0.323, *P* = 0.003). Meanwhile, baPWV negatively correlated with the height of RA patients (*r* = −0.253, *P* = 0.043). However, no significant correlation was found between baPWV and RA-related factors, such as DAS28, RF, and anti-CCP.

### 3.5. Multivariate Regression Analysis of Determinants of baPWV

In a stepwise multiple linear regression analysis, we employed baPWV value as dependent variable while using age, height, cholesterol, triglycerides, and LDL as independent variables.  Table 5 shows the multivariate linear regression analysis results. Age and serum triglycerides level were independently associated with baPWV (*P* < 0.001, *P* = 0.014, resp.). 

## 4. Discussion

Coronary artery disease is typically silent in RA, with a greater frequency of unrecognized myocardial infarction and sudden cardiac death [15, 16]. RA patients have up to 3 times increased standard mortality rates in comparison to the general population, and today cardiovascular diseases (CVD) are the leading cause of death [17–19]. Vascular disease may account for one-third of all deaths in RA [20]. Early detection of arterial stiffness is useful in primary and secondary prevention of series of major CVD [21]. Several studies have shown that PWV was an independent predictor of future development of CVD [22–24]. The baPWV measurement is noninvasive and convenient and has been used for cardiovascular risk stratification [25, 26]. For the first time, the present study investigated the baPWV level and sought to identify the RA-related and the traditional risk factors for CVD in Chinese patients with RA.

The mean baPWV of our RA patients was 1705.44 ± 429.20 cm/s. The value is significant higher than that of matched control (1386.23 ± 411.09 cm/s) (*P* < 0.001). Wang and coworkers measured baPWV in 2852 participants of North China and found that the mean baPWV of healthy sample is 1339.3 ± 224.8 cm/s for male and 1294.8 ± 241.9 cm/s for female [27]. This is similar to the result of our study. It also demonstrated that RA patients in our study had significant higher baPWV value than healthy controls.

In a univariate model, baPWV in RA patients did not show any significant correlation with RA-related factors. These factors included disease duration, RF-IgG, RF-IgA, RF-IgM, anti-CCP, DAS28, ESR, and CRP. The same results were obtained from the comparisons between two subgroups of RA patients. Kim and associates evaluated the baPWV in Korean patients with RA and also found that the baPWV value of RA patients did not correlate with RA-related factors [28]. Contrary to our findings, Mäki-Petäjä and coworkers found that PWV of RA patients correlated independently with log-transformed CRP (*P* < 0.001). The patients in the study of Mäki-Petäjä and coworkers had a mean age of 57 years, while our patients had a mean age of 50 years [29]. The baPWV is known to increase steeply after age of 50 years [30, 31]. It might be the major reason that caused the difference between the results of Mäki-Petäjä study and ours. Moreover, other factors might also result in the difference, such as the Mäki-Petäjä study that only enrolled 77 RA patients, which means the sample size of their study was smaller than ours; and the mean disease duration of their study was 13 years, which was much longer than ours (24 months); on the other hand, the race of participants was totally different between their study and ours.

In this study, the identified potential risk factors of arterial stiffness based on baPWV measurement were older age and higher serum triglycerides level. It is well known that in elder individuals, arteries are less elastic which induces higher velocity of pulse wave [32–34]. Several studies supported that the detection of increase of PWV is parallel to advanced age [35–38]. In the regression analysis of our study, age was found to be the most important independent variable. Ai and coworkers found that serum level of triglycerides was independent predictor of baPWV in Chinese population [39]. It was similar to our results. The patients' age, as one of the independent associated factors with baPWV, cannot be modified. So the attention should be placed on another modifiable risk factor-serum level of triglycerides. Keeping a normal level of triglycerides should be very important for Chinese RA patients avoiding CVD.

Several limitations of this study must be taken into consideration when interpreting the data. Firstly, the cross-sectional design of our study that resulted from the prognostic significance of baPWV could not be evaluated. Secondly, lack of cardiovascular prognosis factors was also a fact in this study. For better understanding of risk factors of arterial stiffness and cardiovascular risk in Chinese RA patients, longitudinal studies including factors of cardiovascular prognosis are necessary.

## 5. Conclusions

In summary, this is the first study describing the factors influencing baPWV in Chinese patients with RA. We have shown that RA is associated with increased arterial stiffness in comparison with healthy control subjects. The arterial stiffness, which was assessed by baPWV, had an independent correlation with age and serum triglycerides level. These are undoubtedly important for preventing CVD of Chinese patients with RA. 

## Figures and Tables

**Table 1:** Clinical characteristics of patients and controls.

Characteristic	RA (*n* = 138)	Controls (*n* = 150)	*P *
Age (years), mean ± SD	50.17 ± 12.49	49.61 ± 8.74	0.500
Female, *n* (%)	111 (80.43)	119 (79.33)	0.816
Weight (kg), mean ± SD	58.74 ± 10.26	59.11 ± 6.09	0.091
Height (cm), mean ± SD	161.77 ± 6.15	163.05 ± 9.12	0.253
BMI (kg/m^2^), mean ± SD	22.41 ± 0.72	22.25 ± 0.09	0.549
SBP (mmHg), mean ± SD	116.57 ± 13.38	114.19 ± 12.58	0.137
DBP (mmHg), mean ± SD	75.59 ± 9.25	74.33 ± 8.26	0.382
Cholesterol (mmol/L), mean ± SD	4.82 ± 0.41	5.15 ± 0.79	0.097
Triglycerides (mmol/L), mean ± SD	1.96 ± 0.96	1.27 ± 0.96	0.025
HDL (mmol/L), mean ± SD	1.03 ± 0.27	1.05 ± 0.22	0.608
LDL (mmol/L), mean ± SD	2.78 ± 0.63	3.16 ± 0.71	0.079
Glucose (mmol/L), mean ± SD	5.46 ± 0.59	5.31 ± 0.57	0.774
baPWV (cm/s), mean ± SD	1705.44 ± 429.20	1386.23 ± 411.09	<0.001

Data are given as mean ± SD or number (and percentage). BMI: body mass index; SBP: systolic blood pressure; DBP: diastolic blood pressure; HDL: high-density lipoprotein; LDL: low-density lipoprotein.

**Table 2:** Disease-related factors in RA patients.

	All patients *n* = 138
Disease duration (months), mean (IQR)	24.00 (10.00–102.00)
RF-IgG positive, *n* (%)	80 (57.97)
RF-IgA positive, *n* (%)	65 (47.10)
RF-IgM positive, *n* (%)	108 (78.26)
Anti-CCP positive, *n* (%)	98 (71.01)
ESR (mm/h), mean ± SD	45.69 ± 21.26
CRP (mg/dL), mean (IQR)	14.90 (5.57–32.55)
DAS28, mean ± SD	5.08 ± 1.00
Currently using DMARDs, *n* (%)	9 (6.52)
Currently using NSAIDs, *n* (%)	74 (53.62)
Currently using corticosteroid, *n* (%)	0 (0)

Data are expressed as mean ± SD when normally distributed or as median (25–75%) when nonnormally distributed. IQR: interquartile range; RF: rheumatoid factor; anti-CCP: anticyclic citrullinated peptide; DAS28: disease activity score for 28 joint indices; ESR: erythrocyte sedimentation rate; CRP: C-reactive protein; DMARDs: disease-modifying antirheumatic drugs; NSAIDs: nonsteroidal anti-inflammatory drugs.

**Table 3:** Demographic and clinical characteristics of RA patients and comparisons between subgroups.

Variables	Low baPWV group (*n* = 76)	High baPWV group (*n* = 62)	*P *
Age (years), mean ± SD	46.67 ± 11.54	55.07 ± 11.86	0.008
Female, *n* (%)	60 (78.95)	51 (82.26)	0.626
Weight (kg), mean ± SD	59.25 ± 5.13	57.96 ± 9.55	0.325
Height (cm), mean ± SD	162.95 ± 6.26	160.69 ± 5.20	0.033
BMI (kg/m^2^), mean ± SD	22.78 ± 2.96	22.27 ± 3.91	0.389
SBP (mmHg), mean ± SD	115.00 ± 13.74	117.04 ± 22.56	0.084
DBP (mmHg), mean ± SD	74.91 ± 9.55	76.07 ± 14.68	0.215
Cholesterol (mmol/L), mean ± SD	4.63 ± 0.79	5.13 ± 0.99	0.035
Triglycerides (mmol/L), mean ± SD	1.79 ± 0.75	2.28 ± 0.68	0.004
HDL (mmol/L), mean ± SD	1.05 ± 2.35	1.01 ± 0.29	0.538
LDL (mmol/L), mean ± SD	2.56 ± 0.65	3.09 ± 0.77	0.006
Glucose (mmol/L), mean ± SD	5.51 ± 0.56	5.34 ± 0.69	0.434
Disease duration (months), mean (IQR)	24 (8, 66)	48 (12, 132)	0.169
RF-IgG positive, *n* (%)	46 (60.53)	34 (54.84)	0.501
RF-IgA positive, *n* (%)	37 (48.68)	28 (45.16)	0.100
RF-IgM positive, *n* (%)	56 (73.68)	52 (83.87)	0.149
Anti-CCP positive, *n* (%)	55 (72.37)	43 (69.35)	0.698
ESR (mm/h), mean ± SD	42.56 ± 23.16	49.74 ± 17.11	0.172
CRP (mg/dL), mean (IQR)	13.90 (5.34, 28.55)	18.15 (6.24, 68.05)	0.447
DAS28, mean ± SD	4.87 ± 1.05	5.26 ± 0.75	0.113
Currently using DMARDs, *n* (%)	5 (6.58)	4 (6.45)	0.976
Currently using NSAIDs, *n* (%)	38 (50.00)	36 (58.06)	0.345

Data are given as mean ± SD when normally distributed, as median (25–75%) when nonnormally distributed, or as number (and percentage). BMI: body mass index; SBP: systolic blood pressure; DBP: diastolic blood pressure; HDL: high-density lipoprotein; LDL: low-density lipoprotein; RF: rheumatoid factor; anti-CCP: anticyclic citrullinated peptide; ESR: erythrocyte sedimentation rate; CRP: C-reactive protein; DAS28: disease activity score for 28 joint indices; IQR: interquartile range; DMARDs: disease-modifying antirheumatic drugs; NSAIDs: nonsteroidal anti-inflammatory drugs.

**Table 4:** The correlations between baPWV and other variables.

Parameters	baPWV	
	*r *	*P *
Age (y)	0.439	<0.001
Disease duration (m)	0.159	0.224
Weight (kg)	0.029	0.831
Height (m)	−0.253	0.043
BMI (kg/m^2^)	0.108	0.249
SBP (mmHg)	−0.007	0.958
DBP (mmHg)	−0.090	0.508
Cholesterol (mmol/L)	0.231	0.035
Triglycerides (mmol/L)	0.293	<0.001
HDL (mmol/L)	−0.139	0.293
LDL (mmol/L)	0.323	0.003
Glucose (mmol/L)	0.181	0.153
RF-IgG (IU/mL)	−0.066	0.495
RF-IgA (IU/mL)	−0.008	0.938
RF-IgM (IU/mL)	−0.089	0.341
Anti-CCP (RU/mL)	−0.064	0.486
DAS28	0.062	0.483
ESR (mm/h)	0.037	0.651
CRP (mg/L)	0.021	0.809

BMI: body mass index; SBP: systolic blood pressure; DBP: diastolic blood pressure; HDL: high-density lipoprotein; LDL: low-density lipoprotein; RF: rheumatoid factor; anti-CCP: anticyclic citrullinated peptide; DAS28: disease activity score for 28 joint indices; ESR: erythrocyte sedimentation rate; CRP: C-reactive protein.

**Table 5:** Multiple linear regression analysis of factors associated with baPWV.

	B	SE	Standardized coefficient	*t *	*P *
Constant	914.17	215.83		4.23	<0.001
Age	15.82	4.17	0.449	3.80	<0.001
Triglycerides	26.78	16.82	0.236	1.86	0.014
